# Targeting Polyamine Oxidase to Prevent Excitotoxicity-Induced Retinal Neurodegeneration

**DOI:** 10.3389/fnins.2018.00956

**Published:** 2019-01-10

**Authors:** Prahalathan Pichavaram, Chithra Devi Palani, Chintan Patel, Zhimin Xu, Esraa Shosha, Abdelrahman Y. Fouda, Ruth B. Caldwell, Subhadra Priya Narayanan

**Affiliations:** ^1^Vision Discovery Institute, Augusta University, Augusta, GA, United States; ^2^College of Allied Health Sciences, Augusta University, Augusta, GA, United States; ^3^Vascular Biology Center, Augusta University, Augusta, GA, United States; ^4^Clinical and Experimental Therapeutics, College of Pharmacy, University of Georgia, Augusta, GA, United States; ^5^VA Medical Center, Augusta, GA, United States

**Keywords:** retina, neurodegeneration, excitotoxicity, polyamine oxidase, MDL 72527

## Abstract

Dysfunction of retinal neurons is a major cause of vision impairment in blinding diseases that affect children and adults worldwide. Cellular damage resulting from polyamine catabolism has been demonstrated to be a major player in many neurodegenerative conditions. We have previously shown that inhibition of polyamine oxidase (PAO) using MDL 72527 significantly reduced retinal neurodegeneration and cell death signaling pathways in hyperoxia-mediated retinopathy. In the present study, we investigated the impact of PAO inhibition in limiting retinal neurodegeneration in a model of NMDA (*N-Methyl-D-aspartate*)-induced excitotoxicity. Adult mice (8–10 weeks old) were given intravitreal injections (20 nmoles) of NMDA or NMLA (*N-Methyl-L-aspartate*, control). Intraperitoneal injection of MDL 72527 (40 mg/kg body weight/day) or vehicle (normal saline) was given 24 h before NMDA or NMLA treatment and continued until the animals were sacrificed (varied from 1 to 7 days). Analyses of retinal ganglion cell (RGC) layer cell survival was performed on retinal flatmounts. Retinal cryostat sections were prepared for immunostaining, TUNEL assay and retinal thickness measurements. Fresh frozen retinal samples were used for Western blotting analysis. A marked decrease in the neuronal survival in the RGC layer was observed in NMDA treated retinas compared to their NMLA treated controls, as studied by NeuN immunostaining of retinal flatmounts. Treatment with MDL 72527 significantly improved survival of NeuN positive cells in the NMDA treated retinas. Excitotoxicity induced neurodegeneration was also demonstrated by reduced levels of synaptophysin and degeneration of inner retinal neurons in NMDA treated retinas compared to controls. TUNEL labeling studies showed increased cell death in the NMDA treated retinas. However, treatment with MDL 72527 markedly reduced these changes. Analysis of signaling pathways during excitotoxic injury revealed the downregulation of pro-survival signaling molecules p-ERK and p-Akt, and the upregulation of a pro-apoptotic molecule BID, which were normalized with PAO inhibition. Our data demonstrate that inhibition of polyamine oxidase blocks NMDA-induced retinal neurodegeneration and promotes cell survival, thus offering a new therapeutic target for retinal neurodegenerative disease conditions.

## Introduction

Neuronal injury in the retina is considered as a major cause of vision impairment in blinding diseases such as glaucoma (Vidal-Sanz et al., 2015; Nucci et al., 2016) and diabetic retinopathy (Yu et al., 2008; Jindal, 2015; Araszkiewicz and Zozulinska-Ziolkiewicz, 2016). Neurons in the ganglion cell layer and inner nuclear layer are affected by these pathologies. Glutamate excitotoxicity is considered as one of the mechanisms of neurodegeneration in the central nervous system, including the retina (Fahrenthold et al., 2018; Sone et al., 2018). NMDA (N-Methyl-D-Aspartate) mediated neuronal damage is a well-established model for studying mechanisms of retinal neuroprotection (Laabich and Cooper, 2000; Pernet et al., 2007; Takeda et al., 2007). It is characterized by the excessive synaptic release of glutamate, which in turn activates post-synaptic glutamate receptors and mediates excitotoxic cell death. The NMDA receptor (NMDAR) subtypes play a major role in this process, mainly by increasing intracellular calcium (Ca^2+^). Activation of NMDAR has been shown to promote neuronal death in the retina (Shen et al., 2006; Chintala et al., 2015).

Polyamines (spermine, spermidine, and putrescine) are involved in various cellular functions, such as cell growth and proliferation (Casero and Pegg, 2009; Weiger and Hermann, 2014). However, dysregulation of polyamine catabolism is associated with various neurodegenerative disease states. Altered levels of polyamines have been implicated in neurological disease conditions such as Alzheimer's disease (Morrison and Kish, 1995; Yatin et al., 2001; Inoue et al., 2013) Parkinson's disease (Gomes-Trolin et al., 2002; Lewandowski et al., 2010; Paik et al., 2010) traumatic brain injury (Zahedi et al., 2010) and in the pathogenesis of ischemic brain damage (Ivanova et al., 2002; Takano et al., 2005; Wood et al., 2006). Even though these results suggest that polyamines play an important role in neurodegeneration, the mechanisms whereby they participate in neuronal death have yet to be elucidated.

Polyamine metabolism is finely regulated by the concerted actions of various enzymes (Casero and Pegg, 2009; Hussain et al., 2017). Spermine Oxidase (SMO), Spermine Spermidine Acetyl Transferase (SSAT), and Acetyl Polyamine Oxidase (APAO) are major enzymes regulating the backward oxidation of the polyamines-spermine, spermidine, and putrescine. Activation of polyamine oxidases (PAOs) including SMO and APAO generates toxic aldehydes and hydrogen peroxide (H_2_O_2_) as byproducts and leads to damage of DNA, RNA, proteins, and lipids (Seiler, 2000; Amendola et al., 2005; Casero and Pegg, 2009). PAOs are the key enzymes in polyamine catabolism, playing an essential role in maintaining polyamine homeostasis (Seiler, 2004; Cervelli et al., 2012). Increasing evidence shows the involvement of PAOs in neurodegenerative diseases (Cervelli et al., 2012; Capone et al., 2013).

MDL 72527 (N,N'-Bis(2,3-butadienyl)-1,4-butanediamine dihydrochloride) is an irreversible competitive inhibitor of the polyamine oxidases (Dogan et al., 1999b; Seiler et al., 2002; Cervelli et al., 2013b). Studies in a rat model of cerebral ischemia have shown that treatment with MDL 72527 significantly reduced brain edema, ischemic injury volume, and polyamine levels (Dogan et al., 1999b). These results are consistent with studies on brain injury models, in which MDL 72527 treatment improved neuronal survival (Dogan et al., 1999a; Liu et al., 2001). Furthermore, blockade of polyamine oxidation using MDL 72527 was found to be protective against edema and necrotic cavitation after traumatic brain injury (Dogan et al., 1999a).

Studies from our laboratory are the first to demonstrate the role of polyamine metabolism in retinal neurodegeneration (Narayanan et al., 2014). Using the mouse model of oxygen-induced retinopathy (OIR), we showed that hyperoxia-induced neuronal degeneration is associated with significant increases in PAO along with altered levels of polyamines, suggesting a significant role for polyamine oxidase in the neuronal injury (Narayanan et al., 2014). Further analysis showed for the first time that treatment with MDL 72527 limits the hyperoxia-induced oxidative stress and reduces retinal neurodegeneration, confirming the role of PAO in neurotoxicity (Narayanan et al., 2014). The role of polyamines and polyamine oxidases in excitotoxicity-induced neuronal damage in the retina is not well-studied. In the present study, utilizing the well-established NMDA model of retinal neuronal injury, we investigated whether treatment with the PAO inhibitor, MDL 72527 reduces excitotoxicity-induced neurodegeneration and its potential as a therapeutic target for retinal neurodegenerative diseases.

## Materials and Methods

### Animals

All procedures with animals were performed by the ARVO Statement for the Use of Animals in Ophthalmic and Vision Research and were approved by the institutional animal care and use committee (Animal Welfare Assurance no. A3307–01) adhered to the Public Health Service Policy on Humane Care and Use of Laboratory Animals (revised July 2017). These studies were conducted using wild-type male C57BL6J mice (8–10 weeks, Jackson Laboratories, Bar Harbor, ME) and all efforts were made to minimize suffering during experimental procedures performed.

### Intravitreal Treatment of NMDA

Retinal excitotoxicity was induced using NMDA (N-Methyl-D-Aspartate, Sigma) according to the published methods with minor modifications (Tsutsumi et al., 2016; Ishimaru et al., 2017). Briefly, mice were anesthetized with ketamine/xylazine (73 mg/kg ketamine hydrochloride and 7.3 mg/kg xylaxine hydrochloride, i.p., intraperitoneally), pupils were dilated with 1% tropicamide (Akorn, Lake Forest, IL, United States), and topical anesthesia (1 drop of proparacaine hydrochloride, Akorn) was applied to the cornea. Intravitreal injection of NMDA (20 nmoles/eye, 1 μl, dissolved in saline) was performed in the right eye using a WPI microsyringe. The needle was kept for additional 30 s and pulled out slowly, and antibiotic ointment was applied. For the control group, NMLA (N-Methyl L Aspartate, 20 nmoles/left eye) was used.

### MDL Treatment

Treatment with PAO inhibitor was performed as described previously (Narayanan et al., 2011; Patel et al., 2016). Briefly, intraperitoneal injection of MDL 72527 (40 mg/kg body weight/day) or vehicle (normal saline) was given 24 h before NMDA or NMLA treatment and continued until the animals were sacrificed (varied from 1 to 7 days).

### Immunofluorescence Staining

Immunostaining of retinal cryostat sections were performed as described previously. (Patel et al., 2016; Shosha et al., 2016) Eyes were enucleated, fixed in 4% paraformaldehyde for overnight at 4°C, washed in PBS, and cryoprotected in 30% sucrose. Cryostat sections (10 um) were obtained and mounted on glass slides. The sections were permeabilized in 0.05% Triton X-100 for 10 min and blocked in 10% normal goat serum containing 1% BSA for 1 h at room temperature. Followed by blocking, the sections were incubated with primary antibodies [SMO, Synaptophysin, Protein kinase Cα (PKCα), choline acetyl transferase (ChAT), Calbindin and Glial fibillary acidic protein (GFAP)] overnight at 4°C. Next day, they were washed and further incubated with Fluorescein-conjugated secondary antibodies (Life Technologies, Grand Island, NY, United States) for 1 h at room temperature. Finally, they were washed in PBS and covered with mounting medium and DAPI (Vectashield Vector Laboratories, Burlingame, CA, United States). Images of the stained section were obtained using a Zeiss (Thornwood, NY, United States) Axioplan Imager microscope and Zeiss Axiovision 4.8.2 software.

### Retinal Thickness Measurement

Frozen retinal sections from mice treated with NMDA or NMLA, with saline or MDL treatment were used to study the retinal thickness and analyzed as described previously. (Narayanan et al., 2011; Yokota et al., 2011) Retinal cross-sections (10 um) with optic nerve attachment were prepared and the morphology was observed using H and E staining. A minimum of four sections at 20 um apart from each other were used per animal. ImageJ software was used to determine the total retinal or inner nuclear layer (INL) thickness.

### RGC Loss Analysis

Immunostaining on retinal flatmounts was performed as we have described previously (Shosha et al., 2016) in our study. Briefly, eyes were enucleated, fixed in 4% paraformaldehyde overnight at 4°C, washed in PBS, and the retinas were isolated. Retinas were cut toward the optic disc to get a flower shape. The retinal tissue was permeabilized (10% Triton X-100 for 20 min), blocked (10% normal goat serum containing 1% BSA and 0.1% Triton X-100 for 1 h at room temperature), and were incubated in primary antibody (NeuN, Neuronal Nuclei) for 2 h at 37°C. Retinas were washed and incubated in respective secondary antibody overnight at 4°C. Lastly, the retinas were washed in PBS and mounted with mounting medium (Vectashield Vector Laboratories, Burlingame, CA, United States). Retinal flat mounts images were obtained using a confocal microscope (Zeiss LSM 510 META, Thornwood, NY, United States). Quantification of NeuN positive cells in the GCL layer was performed using NIH Image J software as described in the previous report from our laboratory (Yokota et al., 2011). Images were taken from each quadrant of the flat mounted retina at the midperiphery (defined as halfway between the optic nerve head and the outer periphery). This was performed using a 20X objective by counting the number of the consecutive non-overlapping fields from the optic nerve to edge. Five serial images (1 μm apart) of NeuN positive GCL were taken from each region and merged to get projection image for quantification.

### TUNEL Assay

Retinal cryosections were used to study the apoptotic cells by using the TUNEL (Terminal deoxynucleotidyl transferase dUTP nick end labeling) assay, according to the manufacturer's protocol (Fluorescein Insitu Cell death detection kit, Millipore, Billerica, MA, United States). TUNEL positive cells were quantified as previously described (Narayanan et al., 2014). TUNEL based detection of apoptotic cells were performed in retinal cryostat sections (formalin fixed) and counterstained with DAPI. Staining without the TdT enzyme was used as negative control. Quantification of TUNEL-positive cells in retinal cryosections from optic disc to periphery was performed manually using a fluorescent microscope (Zeiss Axioplan 2, Thornwood, NY, United States). The number was multiplied by two to represent total number of TUNEL positive cells per retinal section. A minimum of three sections (20 μm apart) per animal were used.

### Western Blotting

Retinal tissue was homogenized in RIPA buffer (Millipore, Billerica, MA, United States) containing protease inhibitor (Complete Mini) and phosphatase inhibitors (phosSTOP, Roche Applied Science, Indianapolis, IN, United States). Protein concentration was measured using the bicinchoninic acid kit (Pierce, Thermo Scientific) according to manufacturer's instructions. Protein samples (15–20 ug) per lane were separated on SDS-PAGE and electrophoretically transferred to nitrocellulose membrane (Millipore, Billerica, MA, United States), blocked in 5% dry milk in 1X Tris-buffered saline with 0.5% Tween-20 (TBST). Membranes were incubated in primary antibodies overnight at 4°C with phospho Akt, total Akt, phospho ERK1/2, total ERK1/2, Bcl-xL, BID, (Cell Signaling Technology, Danvers, MA, United States) diluted in 2% BSA, or beta-Actin (Sigma). Further, the membranes were washed with TBST and incubated with anti-rabbit or anti-mouse HRP-conjugated secondary antibodies (GE-Healthcare, Piscataway, NJ, United States), washed (TBST) and detected using enhanced chemiluminescence system (GE-Healthcare, Piscataway, NJ, United States). The band intensities were quantified by densitometry using ImageJ software and normalized to loading controls.

### Statistical Analysis

GraphPad Prism 7 (GraphPad Software Inc., La Jolla, CA, United States) was used for statistical analysis. Results were presented as mean ± SD. Statistical analysis was performed using one-way or two-way ANOVA followed by Tukey test for multiple comparisons. In case of single comparison, the Student's *t*-test was applied. *P* ≤ 0.05 was considered statistically significant.

## Results

### Increased Expression of SMO During Retinal Excitotoxicity

**Figure 1:**
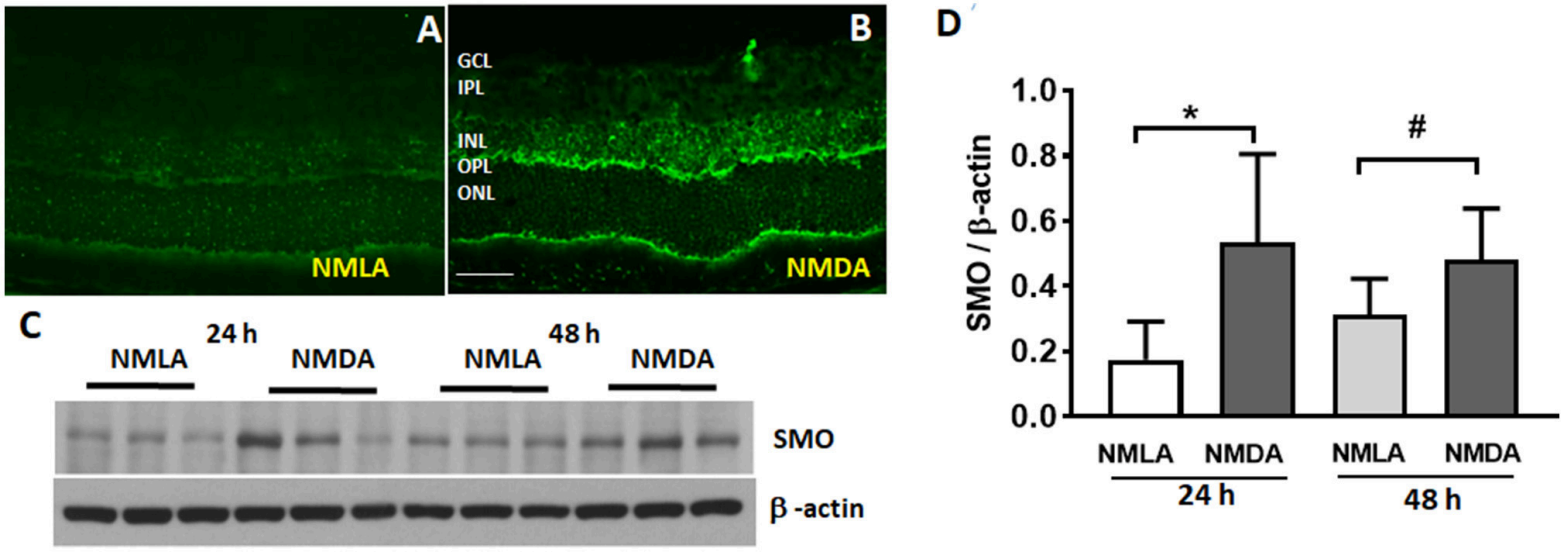
Expression of SMO is increased in response to excitotoxicity. **(A,B)** Immunofluorescence images showing the localization and elevated expression of SMO in the NMDA retinas, 24 h following the injury. **(C)** Western blot studies showing increased SMO levels in NMDA retinas (24 h and 48 h post injury) compared to respective NMLA controls. **(D)** Quantitative analysis show significantly increased (*p* < 0.05) levels of SMO in NMDA retinas relative to respective controls. Data presented as Mean ± SD. ^*^P < 0.01; ^#^*P* < 0.05, *N* = 3–6. Scale bar 50 μm. GCL, ganglion cell layer; INL, inner nuclear layer; IPL, inner plexiform layer; OPL, outer plexiform layer; ONL, outer nuclear layer.

Our previous studies have shown increased SMO levels in the OIR retina (Narayanan et al., 2014). Figure 1 demonstrates the expression and localization of SMO in the retina, in response to the effects of NMDA induced excitotoxicity. Immunofluorescence studies showed the elevated SMO (also called PAO in this study) expression levels in the INL and OPL of NMDA retinas, while the expression was very minimal in NMLA treated controls (Figures 1A,B). Increased expression in SMO was evident in retinal lysates at 24 h and 48 h following NMDA treatment (Figure 1C). Quantitation using Image J analysis shows significantly increased (*p* < 0.05) levels of SMO in NMDA retinas compared to respective NMLA controls at both time points analyzed (Figure 1D). These studies suggest the involvement of SMO in excitotoxicity induced retinal neuronal damage.

### Survival of Retinal Ganglion Cells in Response to MDL Treatment

**Figure 2:**
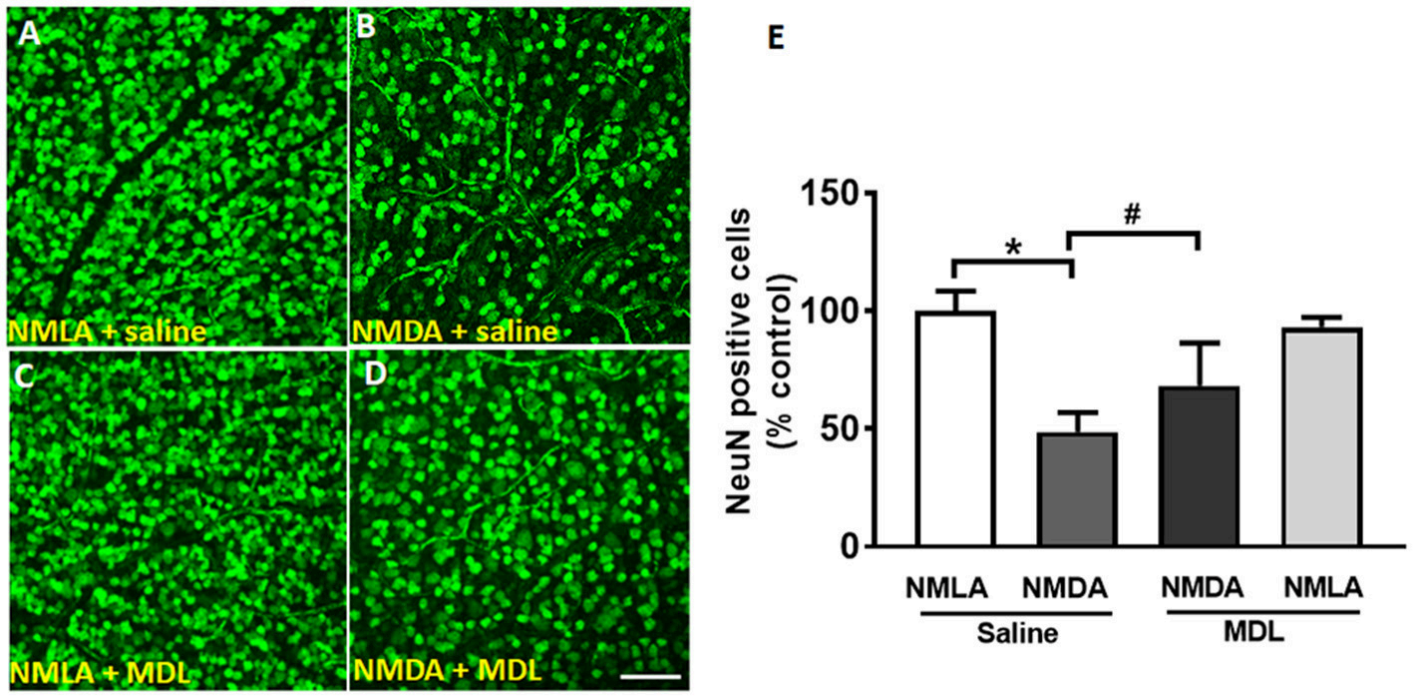
Treatment with MDL reduced excitotoxicity-induced loss of GCL neurons. **(A–D)** Representative confocal images showing NeuN immunolabeling on retinal flat mounts (7 days post-injury) of control (NMLA) or excitotoxic (NMDA) retinas from mice treated with vehicle or MDL. **(E)** Quantitative analysis demonstrating significant loss of NeuN-positive cells in response to excitotoxicity, while MDL treatment improved the survival. Results are presented as a percentage of WT NMLA controls. Data are presented as Mean ± SD. ^*^P < 0.01, ^#^*P* < 0.05. *N* = 4–6. Scale bar 100 μm.

Loss of RGCs is a major feature of retinal excitotoxicity (Tsutsumi et al., 2016; Ishimaru et al., 2017). In the present study, NMDA-induced RGC loss was studied (7 days post-NMDA treatment) using retinal flatmount analysis. Figures 2A–D shows representative confocal images of retinal flatmounts immunostained using NeuN antibody. A marked reduction in the number of NeuN positive cells in the GCL was observed in NMDA retinas compared to their NMLA controls. However, treatment with MDL, the PAO inactivator significantly reduced the excitotoxicity-induced RGC loss. Figure 2E demonstrates the quantitation of the number of NeuN positive cells (RGC layer) in the retinal flatmounts of vehicle or MDL treated NMDA retinas compared to their NMLA retinas. Around 50% reduction in the number of NeuN positive cells (GCL layer) was evident in NMDA retinas compared to NMLA controls (*p* < 0.01). Treatment with MDL significantly increased the number of surviving neurons (70%) in NMDA retinas and significantly increased GCL neuronal survival compared to vehicle-treated NMDA retinas. MDL treatment did not alter RGC survival in the NMLA- treated retinas.

### PAO Inhibition Reduced the Excitotoxicity-Induced Degeneration of Inner Retinal Neurons

**Figure 3:**
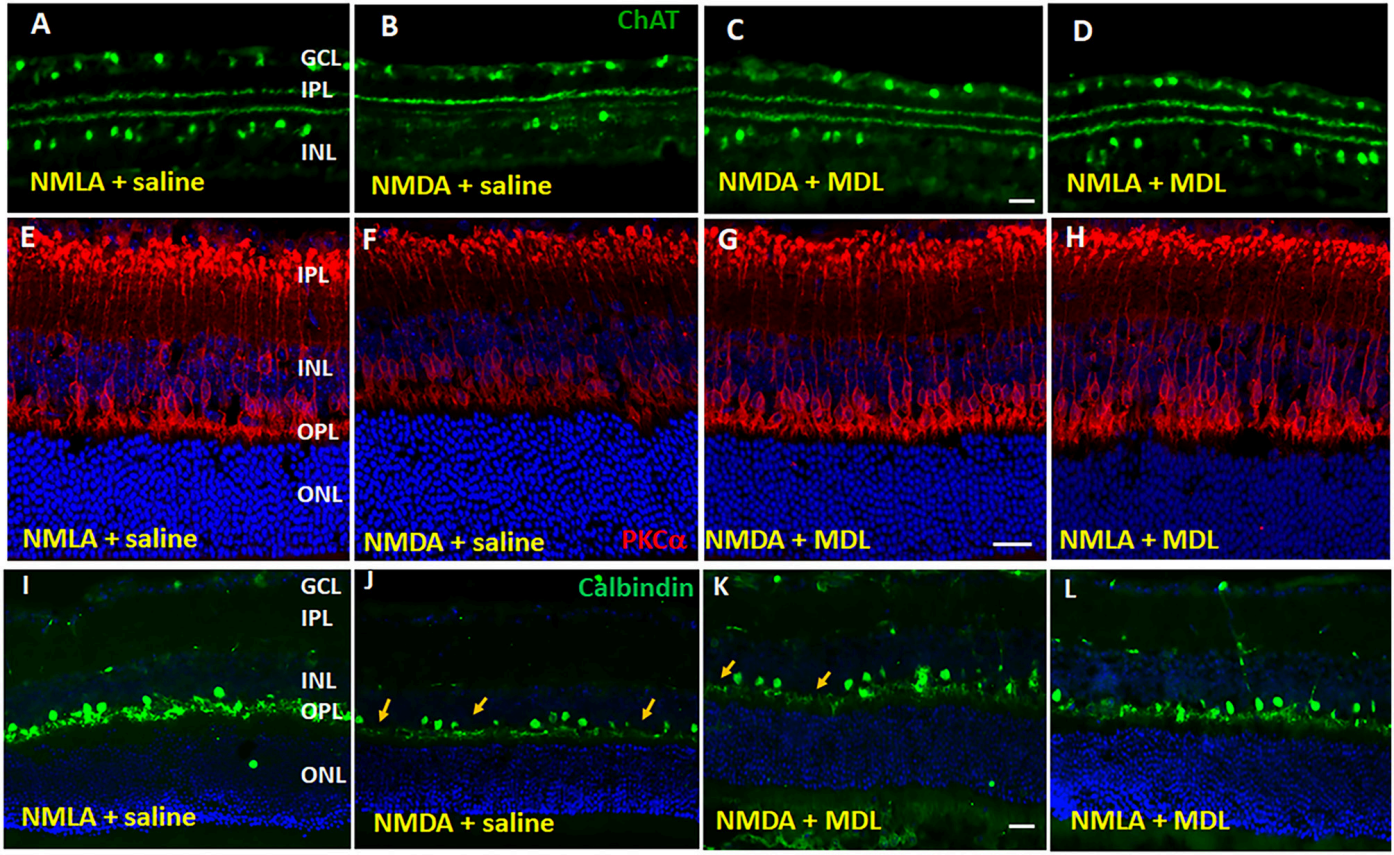
Excitotoxicity-induced neurodegeneration in the inner retina is decreased by PAO inhibition. **(A–D)** Representative confocal images of retinal sections immunolabelled by ChAT, a marker of amacrine cells show reduced number of ChAT positive cells in GCL and INL of NMDA retinas. **(E–H)** Confocal images of retinal sections immunostained by PKCα, a marker for rod bipolar cells, showing the degenerating cells with shorter and distorted dendrites in the NMDA retina. **(I–L)** Calbindin immunolabelling shows the loss of horizontal cells along the OPL of NMDA retinas. Arrows indicate areas of cell loss. Scale bar 50 μm. GCL, ganglion cell layer; INL, inner nuclear layer; IPL, inner plexiform layer; OPL, outer plexiform layer; ONL, outer nuclear layer. *N* = 4–6, and representative images are presented.

Studies have shown that, in addition to RGCs, other retinal neurons are also impacted by NMDA-induced neurotoxicity (Chintala et al., 2015; Kuehn et al., 2017). In our present study, utilizing samples at 5 days post-injury, we investigated whether treatment with MDL improved the survival of neurons in the INL in response to NMDA treatment. Figure 3 demonstrates representative confocal images from retinal sections immunostained using ChAT (Figures 3A–D, the marker for amacrine cells), PKCα Figures 3E–H, the marker for rod bipolar cells), or Calbindin (Figures 3I–L, the marker for horizontal cells). Qualitative analysis showed a trend toward reduction in amacrine cells were observed in NMDA-retinal sections, while treatment with MDL reduced this change. Excitotoxicity induced degeneration of bipolar cells is evident in NMDA retinas (vehicle treated), shown by reduced expression level of the marker, and by the presence of shortened and degenerating dendrites. These alterations were improved in NMDA retinas response to MDL treatment. Calbindin immunostaining showed loss of horizontal cells (in the OPL) in NMDA retinas compared to NMLA controls, while MDL treatment improved the survival of calbindin-positive cells.

**Figure 4:**
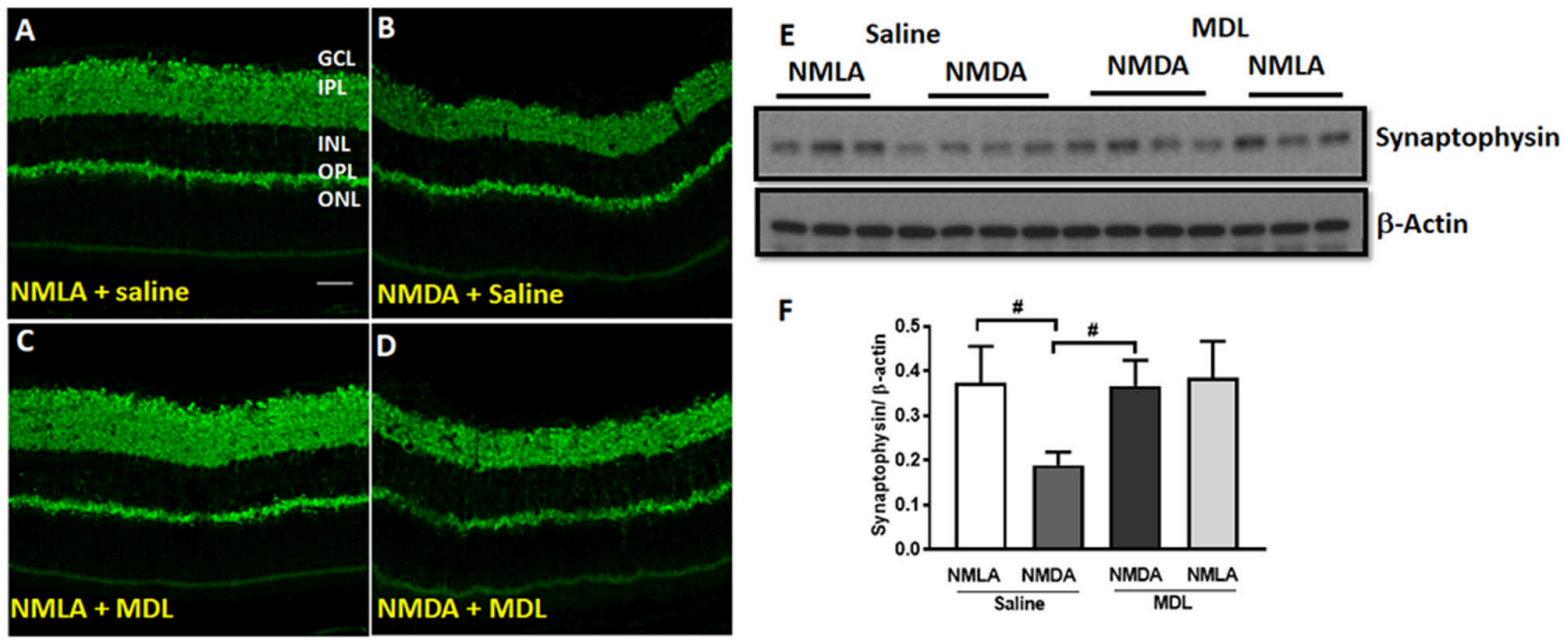
PAO inhibition improved synaptic contacts in NMDA-treated retinas. **(A–D)** Immunofluorescence staining using synaptophysin (a pre-synaptic marker) antibody on retinal sections (5 days post-injury) demonstrating reduced synaptic contacts in NMDA retinas, while MDL treatment increased synaptophysin levels. **(E,F)** Western blot analysis and quantitation showing synaptophysin expression levels in retinal samples. A significant reduction was observed in NMDA-retinal samples, while MDL treatment significantly improved the expression of synaptophysin. Data presented as Mean ± SD. ^#^*P* < 0.05. *N* = 3–4. GCL, ganglion cell layer; IPL, inner plexiform layer; INL, inner nuclear layer; OPL, outer plexiform layer; ONL, outer nuclear layer. Scale bar 50 μm.

Further studies on synaptophysin, a presynaptic marker showed a marked reduction in its level in the NMDA retinas (5 days post-injury), while MDL treatment demonstrated a significant improvement (Figure 4). Confocal images (Figures 4A–D) show reduced expression of synaptophysin in the plexiform layers of NMDA retinas. However, MDL treatment upregulated the synaptophysin levels. Western blot analysis (Figures 4E,F) further confirm these results. Synaptophysin levels were significantly downregulated in NMDA retinas compared to NMLA controls (*p* < 0.05), while MDL treatment resulted in a significant preservation of synaptophysin (*p* < 0.05) levels in NMDA retinas, in comparison with vehicle treated group. These results suggest that PAO inhibition improved the synaptic contacts and reduced the retinal neurodegeneration induced by excitotoxicity.

### Excitotoxicity Induced Retinal Thinning Is Reduced in Response to PAO Inhibition

**Figure 5:**
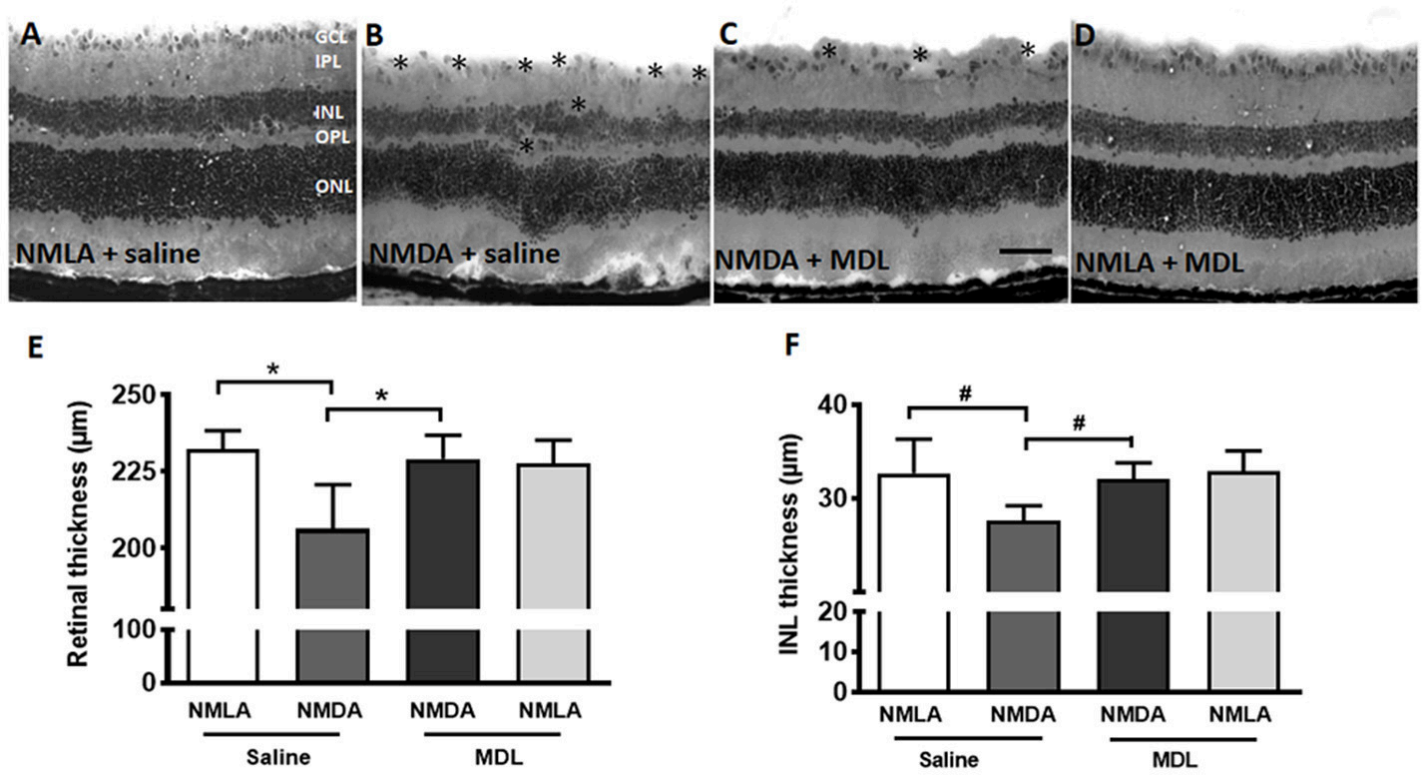
MDL treatment preserved the retinal morphology and thickness. **(A–D)** Representative images of hematoxylin and eosin (H and E) stained retinal sections from NMDA-retinas showing reduced retinal thickness and loss of the cells in the GCL and INL (^*^areas of distortion/missing cells) at 7 days following the injury. This effect of excitotoxicity is markedly reduced in WT mice treated with MDL. **(E,F)** Quantitative analysis showing thickness of retina and INL using retinal sections. A significant reduction of total retinal and INL thickness was observed in the WT NMDA retinas compared to their controls, which was improved in response to MDL treatment. Data presented as Mean ± SD. ^*^P < 0.01, ^#^*P* < 0.05. *N* = 4–6. GCL, ganglion cell layer; IPL, inner plexiform layer; INL, inner nuclear layer; OPL, outer plexiform layer; ONL, outer nuclear layer. Scale bar 100 μm.

Thinning of retinal layers is another characteristic of retinal degeneration (Honjo et al., 2000; Wang et al., 2014; Jindal et al., 2017). In the current study, we investigated whether MDL treatment has an impact on the excitotoxicity induced retinal thinning. Figures 5A–D shows representative images from H and E stained retinal sections from NMDA (7 days post-injury, vehicle or MDL treated) and NMLA (vehicle or MDL treated) controls. Excitotoxicity-induced neuronal damage was evident in the sections from NMDA retinas (7 days post-injury) as seen by degeneration of retinal layers. Loss of cells in the GCL, and a significant reduction in the thickness of total retina (*p* < 0.05) and the INL (*p* < 0.05) thickness were observed in the NMDA (vehicle treated) retinas compared to respective NMLA controls. Treatment with MDL, the PAO inhibitor significantly (*p* < 0.05) protected against the reduction of retinal thickness in the NMDA retinas. Figures 5E,F demonstrates the quantitation of total retinal thickness and INL thickness. The significantly reduced thickness of retina and INL were evident in NMDA retinas compared to the respective NMLA controls. In both cases, a significant increase in the thickness was observed in response to MDL treatment, demonstrating the neuroprotection offered by PAO inhibition.

### Reduced Glial Activation in Response to MDL Treatment

**Figure 6:**
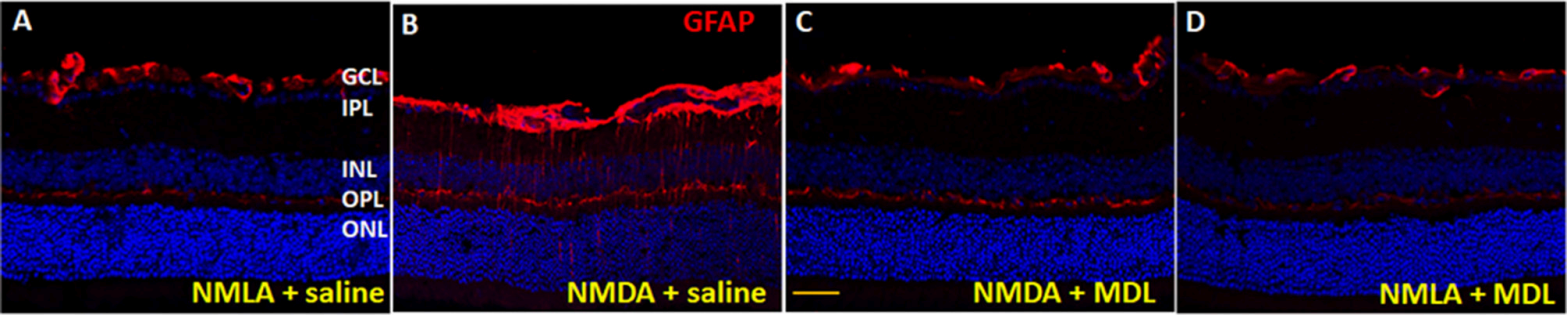
Reduced glial activation in the MDL treated-NMDA retinas. **(A–D)** Immunofluorescence staining of retinal sections (5 days post-injury) using GFAP antibody, demonstrating activation of glial cells in the WT NMDA retina. MDL treatment markedly reduced this effect. *N* = 4–6, and representative images are presented. GCL, ganglion cell layer; IPL, inner plexiform layer; INL, inner nuclear layer; OPL, outer plexiform layer; ONL, outer nuclear layer. Scale bar 100 μm.

Activation of glial cells (studied by GFAP upregulation) in the CNS occurs in response to neuronal injury (Hu et al., 2016). GFAP is typically expressed by astrocytes, and under conditions of retinal stress/injury, Müller cells show elevated GFAP levels. In the present study, glial activation was studied using GFAP immunolabelling of retinal cryostat sections (5 days post injury). Qualitative studies show upregulated levels of GFAP expression in NMDA retinas in comparison with their NMLA controls, while MDL treatment reduced the excitotoxicity mediated glial activation (Figure 6). Our data clearly show the upregulation of GFAP in Muller cells in response to excitotoxicity, as evidenced by increased levels in GCL, IPL, and INL. These changes were abrogated by MDL treatment. These results suggest that inhibition of PAO pathway limited the retinal glial activation in the NMDA-mediated neuronal injury.

### MDL Treatment Reduced the Excitotoxicity-Induced Retinal Cell Death and Cell Death Signaling in the Retina

**Figure 7:**
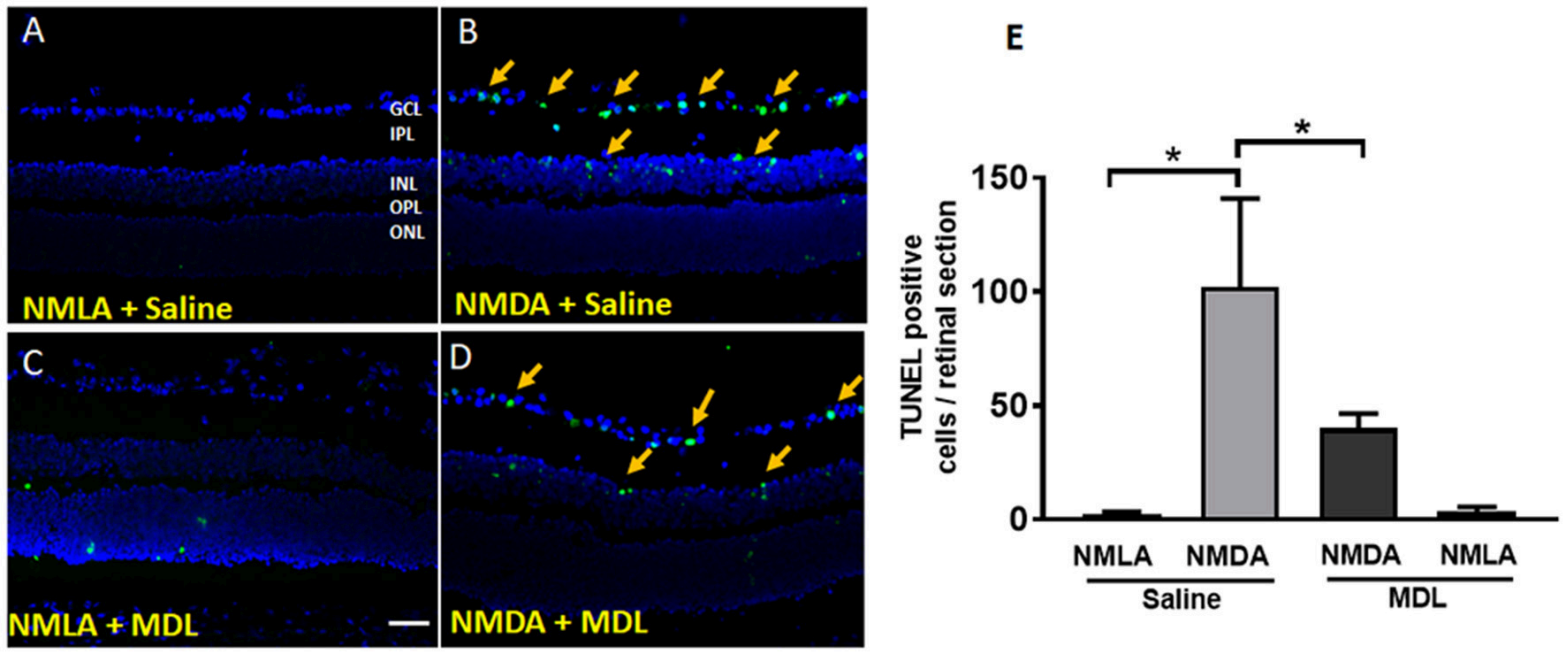
PAO inhibition reduced excitotoxicity-induced cell death. **(A–D)** Representative images showing TUNEL labeling in retinal cryostat sections 3 days following NMDA treatment. The number of cells with fragmented DNA are increased in the WT NMDA retina. PAO inhibition using MDL treatment decreased the number of TUNEL-positive cells in NMDA retinas. Arrows indicate the presence of TUNEL positive cells. **(E)** Quantitation of TUNEL positive cells showing significantly elevated cell death in NMDA retinas, while MDL treatment reduced excitotoxicity-induced retinal cell death. Data presented as Mean ± SD. ^*^P < 0.01. GCL, ganglion cell layer; IPL, inner plexiform layer; INL, inner nuclear layer; OPL, outer plexiform layer; ONL, outer nuclear layer. Scale bar 100 μm.

NMDA induced neurotoxicity leads to retinal cell death. In our study, we analyzed the cell death using TUNEL assay on retinal sections (3 days post injury). As shown in Figures 7A–D, many TUNEL positive cells were present in GCL and INL of NMDA-treated retinas. Inhibition of PAO using MDL treatment significantly reduced the number of TUNEL positive cells. NMLA treatment did not induce any cell death in the retinas studied. Quantitation of the number of TUNEL positive cells in excitotoxic retinas demonstrated a significant decrease in response to MDL treatment (Figure 7E). A significant increase (*p* < 0.01) in the total number of TUNEL positive cells was observed in vehicle-treated NMDA retinas compared to the respective NMLA controls. Treatment with MDL, significantly reduced (*p* < 0.01) the number of TUNEL positive cells in the NMDA retinal sections, in comparison with vehicle treated groups.

**Figure 8:**
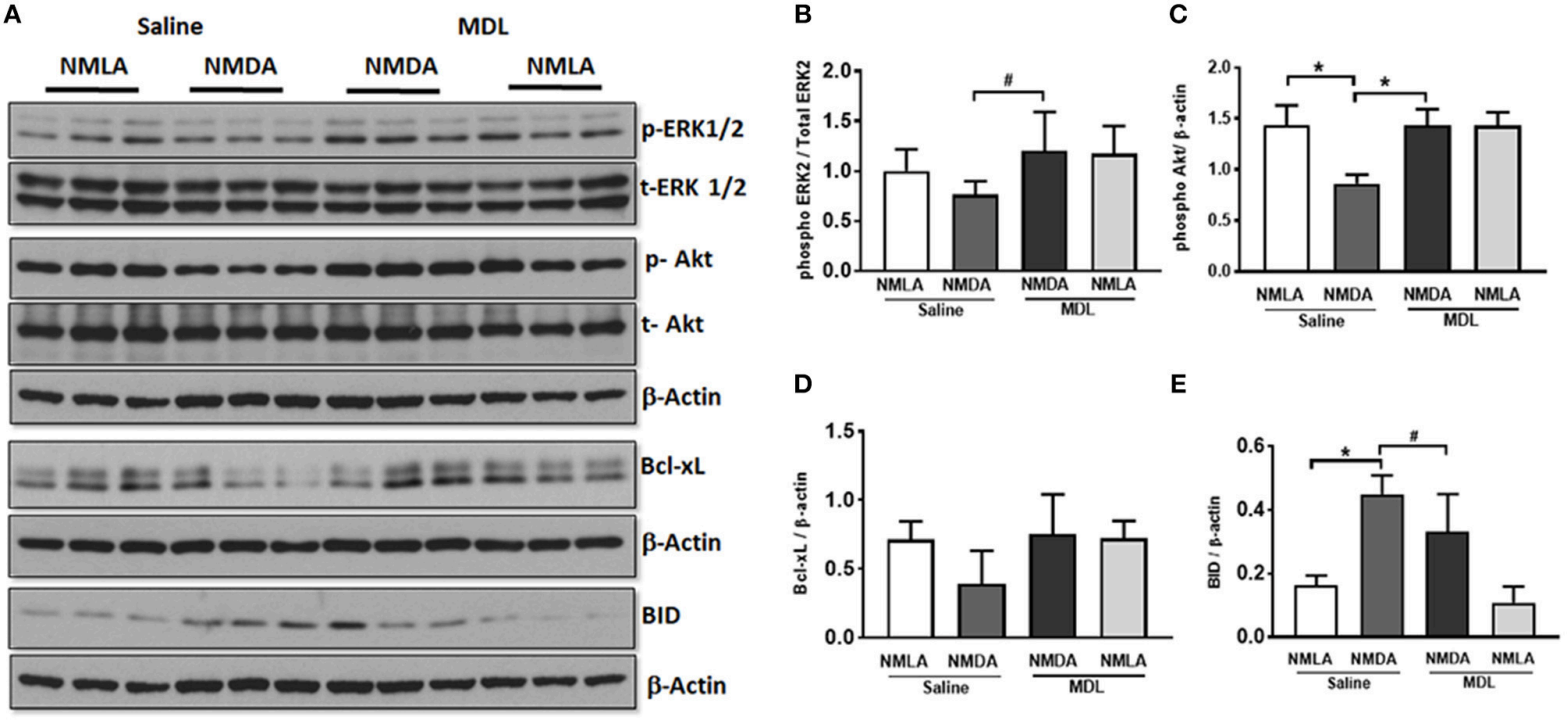
MDL treatment upregulated survival signaling in NMDA retina. **(A)** Analysis of signaling pathways in retinal lysates (48 h post-injury) from control and NMDA retinas treated with vehicle or MDL. **(B–E)** Quantitation of Western blots showing downregulation of the survival pathways along with the upregulation of apoptotic molecules. Treatment with MDL prevented these changes in the NMDA retinas. Data presented as Mean ± SD. ^*^P < 0.01, ^#^*P* < 0.05. *N* = 4–6.

Further studies on the signaling pathways (48 h post-injury) showed downregulation of the survival signals including p-Akt and p-ERK and Bcl-xL in NMDA retinas, while these levels are normalized in response to MDL treatment (Figure 8A). Quantitation of the signaling studies is presented in Figures 8B–E. While there was a tendency toward reduction in the level of p-ERK2 in NMDA retinal samples (compared to NMLA controls), treatment with MDL significantly upregulated p-ERK2 levels in excitotoxic retinas (*p* < 0.05). There was a significant reduction (*p* < 0.01) in the level of p-Akt in NMDA retinas, while MDL treatment increased the level of Akt phosphorylation (*p* < 0.05). A reduction in the expression of total Akt was also evident in NMDA samples hence beta-actin was used to normalize p-Akt levels. A trend in the downregulation of Bcl-xL expression, another survival molecule was observed in retinas treated with NMDA, while the pattern was reversed in response to MDL treatment. However, these changes were not statistically significant. The marked increase in the level of BID, an apoptotic marker in NMDA retinas were significantly reduced (*p* < 0.05) in response to MDL treatment. These studies demonstrate the treatment with MDL, the PAO inactivator significantly reduce excitotoxicity induced retinal cell death and cell death signaling.

## Discussion

The present study provides the first specific evidence for the impact of MDL 72527, the PAO inhibitor in limiting excitotoxicity-induced neurodegeneration in the retina. Retinal excitotoxicity is recognized as a mechanism of neuronal death and dysfunction, and neuronal degeneration during NMDA has been studied previously by several groups (Wang et al., 2014; Ishimaru et al., 2017). However, the molecular mechanisms mediating the process are still not well-understood. Using the mouse OIR model, we have previously shown that polyamine oxidation is increased during hyperoxia-induced retinal degeneration and that treatment with MDL 72527 significantly reduced the retinal cell death in Narayanan et al. (2014) and Patel et al. (2016). In the present study, we are demonstrating neuroprotective effect of MDL 72527 on retinal degeneration in NMDA-induced excitotoxicity. To the best of our knowledge, this is the first report investigating the impact of PAO inhibition in excitotoxicity-induced neurodegeneration in the retina.

MDL 72527 is an irreversible competitive inhibitor of SMO and APAO, commonly known as the polyamine oxidases (Dogan et al., 1999b; Cervelli et al., 2013b). Increased expression or activity of PAO and altered levels of polyamines have been reported in CNS models of neuronal injury (Dogan et al., 1999a; Zahedi et al., 2010; Cervelli et al., 2013a). Studies in a rat model of cerebral ischemia have shown that inhibition of polyamine oxidases using MDL 72527 significantly reduced brain edema, ischemic injury volume, and polyamine levels (Dogan et al., 1999b). Furthermore, blockade of the polyamine oxidation using MDL 72527 was found to be neuroprotective against edema and necrotic cavitation after traumatic brain injury (Dogan et al., 1999a). Our present studies demonstrating increased SMO expression in the inner retina following the retinal damage, is consistent with the earlier studies showing elevated SMO levels in the OIR retina (Narayanan et al., 2014). Elevated SMO/APAO levels indicate increased polyamine oxidation and the resulting oxidative stress (Seiler, 2000).

Loss of ganglion cells is a major feature of excitotoxic injury to the retina. Consistent with other studies (Laabich and Cooper, 2000; Zhao et al., 2016; Ishimaru et al., 2017), we have demonstrated a significant loss of GCL layer neurons following the NMDA treatment. Similar to our previous report on the neuroprotective effect of PAO inhibition in the OIR model (Narayanan et al., 2014), the present studies show that treatment with MDL significantly reduced NMDA-induced neuronal death in the GCL. These results demonstrate the potential neuroprotective effect of MDL 72527 in limiting retinal neuronal injury. There is increasing evidence that in addition to RGC loss, degeneration of other inner retinal neurons, such as bipolar cells and amacrine cells as well as thinning of the INL are observed by NMDA induced retinal damage (Honjo et al., 2000; Kido et al., 2001; Kuehn et al., 2017). In the present study, we observed degeneration of amacrine, bipolar, and horizontal cells in response to NMDA treatment. Furthermore, a significant reduction in the expression of synaptophysin, a pre-synaptic marker was also evident in NMDA-treated retinas. PAO inhibition resulted in an upregulation of synaptophysin and improved survival of bipolar and horizontal cells in the NMDA-treated retinas. Thinning of the retina and retinal layers is another characteristic of neurodegenerative diseases of the eye (Luan et al., 2006; Gupta et al., 2016; Akaiwa et al., 2017; Ortiz et al., 2018). In the current study, a significant thinning of the total retina and INL was evident in the excitotoxic retinas, while treatment with MDL significantly prevented this degeneration, further confirming the neuroprotective effect of PAO inhibition.

Glial activation is considered as an indicator of injury to the retina during disease or stress conditions including ischemia/reperfusion (Yokota et al., 2011; Shosha et al., 2016; Renner et al., 2017), oxygen-induced retinopathy (Narayanan et al., 2011), diabetes (Fernandez-Bueno et al., 2017; Chaurasia et al., 2018) and neurotoxicity by NMDA (Casson et al., 2004; Sakamoto et al., 2017). Although a direct relationship between macroglial activation and ganglion cell death is not well defined, it can be said that Muller cell activation is a feature of retinal neurodegenerative diseases. Increased immunoreactivity for GFAP has widely used as a marker for glial activation. Our data clearly show the upregulation of GFAP in Muller cells in response to excitotoxicity, while MDL treatment abrogated this effect. The present study is the first to demonstrate that the MDL treatment decreases activation of GFAP in the glia during excitotoxicity, suggesting that inhibiting PAO reduces glial damage and limit injury of the inner retinal neurons.

In the present study, TUNEL-positive cells are located in the GCL and INL and clearly increased 3 days following excitotoxic injury, implying the release of factors to activate cell death. Further, our results demonstrate that MDL treatment significantly reduced the number of TUNEL positive cells, indicating that activation of PAO has an important role in the neuronal death process induced by excitotoxicity. A potential link between PAO activity and neuronal death has been described, which showed that inhibiting PAO function with is protective against neurodegeneration in eye disorders. Multiple pathways have been suggested to mediate excitotoxicity induced retinal neuronal death (Fan et al., 2007; Seki and Lipton, 2008; Sakamoto et al., 2016; Fahrenthold et al., 2018). In the present study, we observed downregulation of the survival pathways, Akt and ERK signaling, and upregulation of BID, an apoptotic molecule in the excitotoxic retinas. These changes were prevented in response to PAO inhibition.

Several mechanisms have been proposed responsible for NMDA-induced retinal neuronal death including inflammation and oxidative damage. The downstream mechanisms of PAO mediated excitotoxicity clearly involve oxidative stress. Oxidation of polyamines has been shown to lead to the generation of H_2_O_2_ and the reactive aldehydes (Seiler, 2004). We have previously shown that MDL treatment reduced H_2_O_2_ mediated oxidative damage in the OIR retina (Narayanan et al., 2014; Patel et al., 2016). Overproduction of H_2_O_2_, a mediator of oxidative stress has also been linked to excitotoxic injury in a transgenic mouse model overexpressing SMO in neonatal cortex (Cervelli et al., 2013a). However, the present study has not addressed whether polyamine oxidation causes excitotoxicity-induced cell death via ROS formation in the retina. An earlier study showed that polyamines regulate NMDA induced excitotoxic retinal neuronal death in rats (Pernet et al., 2007). The investigators found that inhibition of polyamine synthesis using DFMO (difluoromethylornithine) was neuroprotective, while administration of putrescine or spermine potentiated NMDA-induced RGC death. However, the specific mechanisms of this excitotoxic neuronal death have not been investigated. We believe that oxidative stress could be one of the potential mediators of polyamine oxidase activity- induced retinal neuronal death.

**Figure 9:**
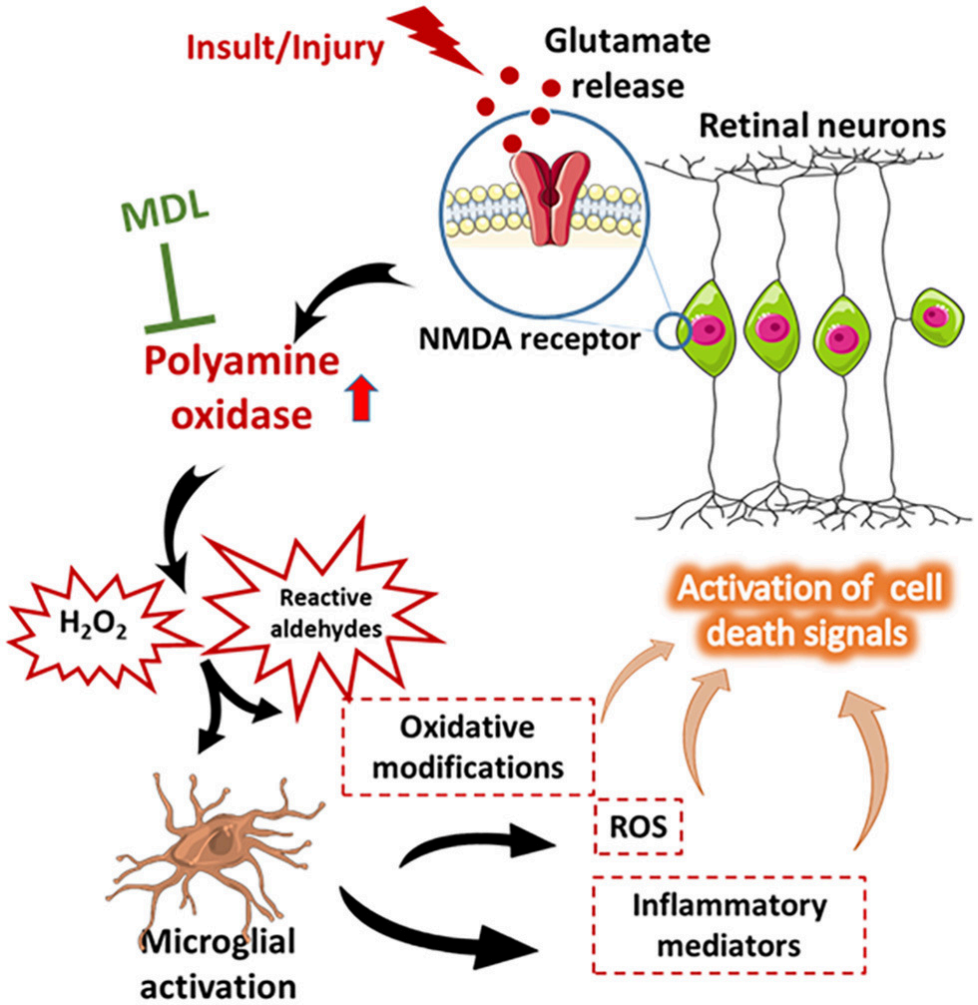
Proposed molecular network of excitotoxicity. A diagrammatic representation of the proposed molecular mechanism of excitotoxicity-induced retinal neuronal death. This figure was prepared using Servier Medical Art (https://smart.servier.com/).

Increasing evidence suggests that microglia, the resident immune cells of the central nervous system are activated in neurological diseases and initiate neuron damage, amplify ongoing neurotoxicity, and drive chronic neuronal loss over time (Block et al., 2007). When over activated, microglia can produce significant and highly detrimental neurotoxic effects by the excess production of array of cytotoxic factors such as superoxide (Colton and Gilbert, 1987; Kaneko et al., 2012; Zhang et al., 2012), nitric oxide (Moss and Bates, 2001; Ribeiro et al., 2013), and tumor necrosis factor-α (Yang et al., 2013; Borrajo et al., 2014; Chen et al., 2014). Reactive microglia have been demonstrated to be present in retinal samples from patients and/or disease models of diabetic retinopathy, glaucoma, ischemia reperfusion injury, and retinopathy of prematurity. Our previous study had shown that OIR-induced microglial activation and generation of inflammatory mediators are significantly reduced in response to PAO inhibition (Patel et al., 2016). Effect of MDL treatment on microglial activation and its impact on excitotoxicity-induced neuronal death will be investigated in our future studies. A proposed molecular mechanism for excitotoxicity-induced retinal neuronal death is presented in Figure 9.

In conclusion, our studies report for the first time the specific impact of inhibiting polyamine oxidation, using MDL 72527 treatment, in limiting excitotoxicity-induced neuronal damage in the retina. We have demonstrated a crucial role for the polyamine oxidase pathway as one of the major mechanisms associated with neuronal injury in the excitotoxic retina. Considering the need for new therapies for patients suffering from vision problems associated with retinal neurodegeneration, such as diabetic retinopathy or glaucoma, our findings are highly relevant from a clinical perspective. Our data suggest that inhibition of polyamine oxidase signaling can be considered as a therapeutic target to limit neuronal dysfunction in neurodegenerative diseases of the eye.

## Author Contributions

PP, CDP, and CP implemented experiments, analyzed the data, assembled the figures, and edited the manuscript. ZX generated the *in vivo* experimental model and helped with analyzing data. ES performed cell death studies and revised manuscript. AF helped analyzing experimental data and revising manuscript. RC contributed toward designing experiments and revising manuscript. SN provided experimental design and planning the entire study, and provided guidance with writing, editing, and revising the manuscript.

### Conflict of Interest Statement

The authors declare that the research was conducted in the absence of any commercial or financial relationships that could be construed as a potential conflict of interest.
